# Medical Societies—Reporting

**Published:** 1830-07-01

**Authors:** 


### MEDICAL SOCIETIES—REPORTING.

No impression, however keen, no sensation, however vivid, can last longer than nine days in the public mind—not even in the grave and sad mind of physic. We have a very recent instance in illustration. We were informed, by a most veracious cotemporary, that an "intense excitement" existed in the professional public on the 15th of April last, respecting the "great public dinner" to Mr. Handey. We think, on reflection, that this must have been the fact—for so intense was the excitement, that it destroyed all appetite before the first of May—and that profession which, according to our cotemporary, was in a complete state of *effervescence* in the middle of the month, became as vapid as stale infusion of senna, before the end of the same month! This is the case with all fervid emotions of the mind, whether in individuals or societies. They cannot last long—like the Irishman's love, they would "burn through the clothes," if they existed beyond the ninth day. Our good friend Wakley should have been aware of this, and not set the profession in a flaming state of excitement for a "great public dinner," fourteen days before the beef was put on the spit! How could he suppose that John Bull's patience would last out such a time, when the pleasure of eating was in view? The "*ensuing Autumn*," however, will make up for all disappointments; and, as the town is then generally in the country, there will be plenty of room in the Freemason's Tavern for the "great public dinner" during the dog-days.

This reminds us of another "intense excitement" that was kindled up, not long ago, respecting reports from the different medical societies. The system of reporting was to introduce a new æra in medical science—to diffuse the knowledge and talents of the metropolitan lions among the humblest members of the profession—to call forth modest and pining merit from the darkest "holes and corners" of our London lanes and alleys—and, in short, to foster talent and to check pretension wherever they were found! These fond speculations have not succeeded. From whatever cause, the fact is, that the attendance on medical societies, in the metropolis, never was at a lower ebb than during the session just terminated. The first effect of reporting was, to call forth a host of candidates for the honor of appearing IN PRINT—and especially those who, of all others, were least capable of affording useful information to the members of societies or the public at large. The next and natural effect was, a cessation of attendance on the part of those who were most likely to render discussions interesting or useful; and the third consequence was, a disappointment of those splendid results of "REPORTING," which the "REPORTERS" of sanguine temperaments had anticipated. Our readers must have seen that the reports of the last session exhibited a sad falling off, and were little more than professional puffs which had outlived their ingenuity and died a natural death.

Our attention was drawn to the subject by reading, in the last Number of our Glasgow cotemporary, certain stormy proceedings in the medical society of that flourishing city, respecting the propriety of "REPORTING" their proceedings. It appears that our cotemporary has got into a scrape by following the routine of the day, and attempting to fix or freeze the wordy thoughts of the Glasgow lions, and thus to give them "a local habitation and a name." The members of the northern congregation do not at all assimilate with those of the south. So far from fighting for publicity, and going on their bended knees to REPORTERS, to beg for a niche in the TEMPLE of FAME, they are said to have viewed, "with feelings of indignation and astonishment," the attempt that was laudably made by our cotemporary to transmit their names to posterity.

"It was then stated, 'with feelings of indignation and astonishment,' that a gross breach of confidence had been committed by some member or members; that the laws had been violated; that the Society had been betrayed, and was about to be utterly ruined; in a word, that the Proceedings of the Society had been reported in the Glasgow Medical Journal, an unprincipled publication, *not a whit better than the Lancet*."

Now we must declare that this last insinuation is utterly devoid of foundation. We have regularly perused the pages of the Glasgow Journal, from its commencement, and we never saw any thing like illiberality or party-feeling in its columns. Indeed it formed a complete contrast to the LANCET, with which it is compared on this point; and, therefore, we cannot but suspect that the above declaration was founded on those very *personal feelings and motives* which are decried in the proceedings of the society. In the course of an angry discussion on the subject of "REPORTING," our cotemporary affirms that a final majority was in favour of the measure; and, consequently, that the Editors of the Glasgow Journal might have fairly and honourably pursued the course which they commenced. But, unlike some of their cotemporaries, they have modestly deferred to the *minority*—and chose to sacrifice their own interests, and even what they conceive to be the interests of the profession, rather than offend the feelings of any members of the society. This procedure alone is a proof of the falsity of the parallel which was attempted to be drawn between the Glasgow Journal and the Lancet. We wish we could verify, *to the full extent*, the following flattering picture of medical societies in general.

"An assemblage of medical men, however, is of all others the least apt to be led away by mere declamation. Habituated, by daily practice, to the command of their feelings, and to seek, even in the most perplexing circumstances, for some sure principle on which to rest their conclusions, their minds acquire a hardihood, which renders them little susceptible of momentary excitement, and a decision, which enables them to view every question in its pure practical bearings, and form their judgment without regard to extrinsic considerations."

We believe that, upon all important occasions, when the judgment as well as the feelings of medical societies are fully called forth, the foregoing results will obtain. But we have had too much knowledge of the world, and of medical societies, to acquiesce, *to the full extent*, in the foregoing proposition. In the cold regions of the North, it may be as our Glasgow cotemporary represents it. But, whether it be owing to the brilliancy of the skies, or the heat of the gas, in modern Babylon, it so happens that the judgment is not *always* formed "without regard to extrinsic considerations." We respect the feelings which dictated the following passage, and the resolution therein promulgated, though the sentiments will scarcely bear the scrutiny of rigid and cold philosophy.

"The Glasgow Medical Society consists of individuals, not merely related as members of a medical association, but related still more intimately by friendship, kindred pursuits, and habitual intercourse. The relations of the latter kind we value still more highly than those of the former, and should be sorry to infringe them. Having ascertained, then, that the publication of the reports of the Society is obnoxious to several Gentlemen, whose opinions we respect, even while we differ from them, we, for that reason, abandon our proposed plan. We repeat, however, that we abandon it, solely, from motives of private feeling."

Whoever, after the above declaration, pronounces the Glasgow Journal to be "an unprincipled publication, *not a whit better than the Lancet*," must be totally incapable of distinguishing light from darkness. Indeed, we conceive that our brother editors of the north have carried their delicacy even too far; for, after a majority of a society had determined the *right* of publishing their transactions, there could be no violation of propriety or delicacy in giving a fair and correct exposition of their proceedings. We begin, however, to doubt very much the ultimate utility of these reportings, since they draw with them a host of evils when the medical press is corrupted by party feelings, and, consequently, incapable of giving a fair representation of the facts or opinions broached in a society. By this procedure all well authenticated cases of much interest are necessarily held back, for fear of exposure to the eyes of relations or friends in the public newspapers—for which vehicles matters are now-a-days *got up* in medical journals, and not at all for professional perusal.

